# Automated calculation of background parenchymal enhancement as a biomarker of treatment responses and recurrence-free survival in breast cancer

**DOI:** 10.1007/s10549-026-07941-5

**Published:** 2026-03-24

**Authors:** Yihui Zhu, Roham Hadidchi, Hien Quang Nguyen, Surya Hariharan, Jeremy Weiss, Wei Hou, Chris Chung, Ha Manh Luu, Siddarth Ragupathi, Takouhie Maldjian, Tim Q. Duong

**Affiliations:** 1 Department of Radiology, Albert Einstein College of Medicine and Montefiore Health System, Bronx, NY USA; 2 Department of Radiology, Stanford University School of Medicine, Palo Alto, CA USA; 3 Faculty of Electronic and Telecommunication, VNU University of Engineering and Technology, Hanoi, VN Vietnam

**Keywords:** Neoadjuvant chemotherapy, Progression-free survival, Molecular subtypes, Human epidermal growth factor receptor 2 (HER2) status, Triple negative

## Abstract

**Purpose:**

To determine whether automated quantification of background parenchymal enhancement (BPE) from dynamic contrast-enhanced MRI (DCE-MRI) can serve as an imaging biomarker for clinical outcomes including overall survival (OS), recurrence-free survival (RFS), and pathological complete response (pCR) in breast cancer.

**Methods:**

The multi-institutional data consisted of 922 biopsy-confirmed invasive breast cancer patients from the Duke-Breast-Cancer-MRI dataset and 152 patients with whole-breast pre- (T_0_) and/or post (T_3_) DCE-MRI from the I-SPY2 dataset for validation. Automated fibroglandular tissue (FGT) segmentation and BPE quantification were performed on DCE-MRI. The optimal intensity enhancement threshold by volume-based method was established against four radiologist-defined BPE categories. The area under the curve (AUC) was obtained for classification of BPE categories. Cox proportional hazards models were used to predict OS and RFS. Logistic regression was used to predict pCR.

**Results:**

Peak-contrast BPE showed strong correlation with radiologist-defined BPE, achieving the best performance at a 55% signal enhancement threshold (AUC 0.70–0.86). The calculated BPE decreased after neoadjuvant chemotherapy. A reduction in calculated BPE grade after neoadjuvant chemotherapy was predictive of pCR for the high baseline BPE group (adjusted odds ratio = 5.88 [1.03, 33.33]) and for the low baseline BPE group (adjusted odds ratio = 6.54 [1.26, 33.94]). Baseline BPE was independently associated with improved OS (adjusted hazard ratio 0.58 [0.34, 0.99]) but not associated with RFS.

**Conclusion:**

Automated quantification of BPE from DCE-MRI provides an objective and reproducible imaging biomarker associated with treatment response and overall survival in breast cancer. These results support its potential utility for individualized risk stratification and therapeutic decision-making.

**Supplementary Information:**

The online version contains supplementary material available at 10.1007/s10549-026-07941-5.

## Introduction

Background parenchymal enhancement (BPE)—the degree of contrast uptake in fibroglandular tissue (FGT) derived from dynamic contrast-enhanced magnetic resonance imaging (DCE-MRI) in breast cancer—has emerged as a surrogate marker of hormonal responsiveness, microvascular function, breast cancer risk, treatment response, and prognosis. Higher BPE has been associated with increased breast cancer risk, more aggressive tumor subtypes, and poor treatment response [1–3]. Many studies have utilized radiologist-defined BPE grade to predict treatment response, recurrence-free survival, among others [4–8].

BPE is currently assessed visually by radiologists using breast imaging-reporting and data system (BI-RADS) categories in clinical settings, which have high interobserver variability and limited reproducibility [9]. To overcome these limitations, a few recent studies have investigated automated and quantitative methods to assess BPE based on precise segmentation of FGT and pixel-wise contrast enhancement kinetics [9, 10]. Some of the challenges include fully automated breast and FGT segmentations. Advances in deep learning and image processing methods have enabled robust segmentation of breast and FGT compartments in both pre- and post-contrast images, providing reproducible inputs for objective BPE quantification [11–16].

A critical step in quantitative BPE assessment is the choice of enhancement threshold to distinguish true enhancement from noise or background fluctuations. Prior works have shown that optimizing signal intensity thresholds—typically expressed as percentage increases from pre-contrast baseline—can improve correlation with radiologist-defined BPE and better stratify patients by risk or treatment response [17, 18]. However, prior studies using automated BPE calculation had small sample sizes. Consensus is still lacking on the optimal enhancement thresholds or whether performance varies across imaging sequences, breast laterality, or timing of contrast uptake. Furthermore, the prognostic value of quantitative BPE, particularly in the context of neoadjuvant chemotherapy (NAC), remains underexplored. BPE may reflect dynamic changes in the tumor microenvironment and surrounding stroma in response to therapy, providing a potentially valuable biomarker for treatment efficacy and survival outcomes [3].

In this study, we evaluated an automated pipeline for breast segmentation, FGT segmentation, and quantitative BPE calculation using DCE-MRI. We performed multi-dimensional optimization of the enhancement thresholds for BPE quantification, compared BPE derived from different imaging DCE phases, and assessed its predictive value for overall survival (OS), recurrence-free survival (RFS), and pathological complete response (pCR) on multi-institutional data. Multivariate models were employed to assess the relative predictive values of baseline BPE and changes in BPE at optimal enhancement thresholds compared to known variables (such as age, demographics, tumor stage at diagnosis, and molecular sub-groups, among others).

## Materials and methods

### Patient cohorts

A total of 1074 patients from two open databases were (1) 922 biopsy-confirmed invasive breast cancer patients with DCE-MRI data from the Duke-Breast-Cancer-MRI dataset [19] were used for training and testing, and (2) 152 patients with whole-breast pre-treatment (T_0_) DCE-MRI from the I-SPY2 dataset [20], of which a subset of 114 patients who underwent both pre- and post-neoadjuvant chemotherapy (NAC) DCE-MRI scans (T_0_ and T_3_). Six patients from the I-SPY2 dataset were excluded because their images contained major artifacts or did not capture the full view of both breasts.

### Qualitative BPE grading by radiologists

BPE was scored as grade 1 (minimal), grade 2 (mild), grade 3 (moderate), and grade 4 (marked) based on BI-RADS by two radiologists and one medical student by consensus review under the supervision of a board-certified radiologist with over 20 years of experience in breast cancer imaging. The assessment was based on visual evaluation of enhancement in the subtraction image, generated by subtracting the pre-contrast image from the first post-contrast image. The qualitative BPE grades served as the reference standard for training and validating the automated quantitative BPE calculation model.

### Mask generation

Breast mask and fibroglandular tissue (FGT) mask were generated from DCE-MRI using a 3D U-Net segmentation model [21]. Breast tumor voxels were excluded from BPE calculation using a 3D nnU-Net-based tumor segmentation model [22].

To train the tumor segmentation model, 50 patients from the I-SPY2 dataset and 300 patients from the Duke-Breast-Cancer-MRI dataset were randomly selected for manual segmentation. The tumor masks were segmented based on visual enhancement of DCE-MRI images by a research engineer and a radiologist under the supervision of a board-certified radiologist with over 20 years of experience in breast imaging. Intensity normalization was then applied using z-score transformation. The pre-contrast, first post-contrast, and second post-contrast phases of each preprocessed DCE-MRI scan were used as three input channels into the model. An average dice similarity coefficient (DSC) of 0.84 was achieved for the dataset using fivefold cross-validation.

For patients with unilateral breast cancer, the contralateral (unaffected) breast was used for BPE calculation. In the few patients with bilateral breast tumors, the side with smaller tumor volume was considered the contralateral breast.

Whether BPE could be reliably calculated using normal tissue in the ipsilateral breast was also evaluated.

### Quantitative BPE calculation

The main analysis employed the volume-based method that has been commonly used in prior studies for automated quantitative BPE calculation [23]. The quantitative BPE is defined as follows: $S_{0,j}$ denotes the signal intensity of voxel j on the pre-contrast image, $S_{i,j}$ denotes the signal intensity of the same voxel on the i post-contrast image, and $V_{Breast}$ represents breast volume.

$$ BPE_{i,k} \; = \;\frac{1}{{V_{Breast} }}\mathop \sum \limits_{j \in FGT} \left\{ {V_{FGT} |\left( {\frac{{S_{i,j} - S_{0,j} }}{{S_{0,j} }}} \right) \times 100\% \ge k\% } \right\} $$

### Analysis

The Spearman rank correlation coefficient was used to assess the consistency between qualitative BPE grades and quantitative BPE. The optimal enhancement threshold was determined by identifying the value that yielded the highest correlation with the qualitative BPE grades. This optimal threshold was then applied in subsequent analyses.

To evaluate the ability of quantitative BPE to distinguish between qualitative BPE categories, logistic regression was performed for the following sub-group comparisons: grade 1 vs. grades 2–4, grades 1–2 vs. grades 3–4, and grades 1–3 vs. grade 4. For each comparison, the area under the curve (AUC) was reported. AUC values were computed for BPE measurements derived from the contra- and ipsilateral side. In addition, the impact of using later phases of post-contrast DCE-MRI image for BPE calculation was also evaluated.

To assess longitudinal changes in BPE before and after NAC treatment, a paired *t* test was performed on BPE values calculated from pre- and post-treatment DCE-MRI scans for patients in the I-SPY2 dataset stratified by the four qualitative BPE grades assigned prior to treatment.

In addition, logistic regression was used to determine an ideal threshold to classify patients as BPE grade 1/2 or 3/4. After dichotomization based on quantitative BPE, Cox proportional hazards models were used to assess whether dichotomized quantitative BPE was associated with overall survival (OS) and recurrence-free survival (RFS) in the Duke-Breast-Cancer-MRI dataset. Other variables in the model included age at diagnosis, menopausal status, lymph node invasion, tumor stage, histologic grade, hormone receptor and human epidermal growth factor receptor 2 (HER2) status, and neoadjuvant chemotherapy. Only variables that were significantly associated with outcomes in the univariate analysis were included in the multivariate models. In addition, analysis was stratified by neoadjuvant chemotherapy/primary surgery status, menopausal status, and age older than 52 years old (the cohort’s median).

We also assessed whether baseline BPE at T0 and change in BPE after NAC in the I-SPY2 dataset were associated with pathological complete response (pCR) using logistic regression to calculate odds ratios. Other variables in the model included age at diagnosis, menopausal status, and hormone receptor and HER2 status. Only variables significantly associated with pCR in the univariate analysis were included in the multivariate models. This analysis was stratified by radiologist-scored BPE at T0 (grade 1/2 or grade 3/4). At each time-point (T0 and T4), logistic regression was used to determine quantitative BPE (1, 2, 3, or 4) and the primary predictor was defined as any changes (increase or decrease) in quantitative BPE.

### Statistical analysis

The area under the curve (AUC) was used to evaluate performance for classification of BPE categories. Cox proportional hazards models were used to predict OS and RFS. Logistic regression was used to predict pCR. *P* value < 0.05 was considered statistical significance unless otherwise specified.

## Results

Table 1 provides demographics of patients in each BPE category. Individuals with minimal BPE (grade 1) were on average older and more likely to be post-menopausal than all other BPE categories. Individuals with higher grade BPE trended younger and more likely to be pre-menopausal. Race, ethnicity, and molecular subtypes did not differ in proportion across BPE groups. Baseline demographics of patients in the Duke-Breast-Cancer-MRI cohort only are shown in Supplemental Table 1.

**Table 1:** Baseline demographic characteristics of patients at time of breast cancer diagnosis for the entire cohort. *SD* standard deviation. *IQR* interquartile range. *HR* hormone receptor. *HER2* human epidermal growth factor receptor 2. *p < 0.05, **p < 0.01, and ***p < 0.001 as compared to grade 1

	Qualitative background parenchymal enhancement grade			
	1 (*n* = 471)	2 (*n* = 299)	3 (*n* = 205)	4 (*n* = 99)
Age				
Age, mean ± SD	54.94 ± 11.49	51.61 ± 10.75 ***	49.32 ± 10.50 ***	46.69 ± 10.04 ***
Age, median [IQR]	55.04 [47.90, 62.93]	51.27 [44.58, 59.00] ***	48.44 [42.64, 56.05] ***	45.12 [40.96, 51.01] ***
Race/ethnicity				
White	335 (71.13%)	204 (68.23%)	153 (74.63%)	67 (67.68%)
Black or African American	94 (19.96%)	63 (21.07%)	34 (16.59%)	22 (22.22%)
Asian	10 (2.12%)	8 (2.68%)	4 (1.95%)	2 (2.02%)
Hispanic or Latino	14 (2.97%)	14 (4.68%)	6 (2.93%)	7 (7.07%)
American Indian or Alaska Native	13 (2.76%)	4 (1.34%)	5 (2.44%)	1 (1.01%)
Native Hawaiian or Pacific Islander	1 (0.21%)	2 (0.67%)	1 (0.49%)	0 (0.00%)
Multi-racial	4 (0.85%)	4 (1.34%)	1 (0.49%)	0 (0.00%)
Molecular subtype				
HR + HER2 −	296 (62.85%)	173 (57.86%)	121 (59.02%)	58 (58.59%)
HR + HER2 +	54 (11.46%)	37 (12.37%)	20 (9.76%)	12 (12.12%)
HR − HER2 +	29 (6.16%)	24 (8.03%)	19 (9.27%)	3 (3.03%)
HR − /HER2 − (Triple negative)	92 (19.53%)	65 (21.74%)	45 (21.95%)	26 (26.26%)
Menopausal status				
Premenopausal	157 (33.33%)	149 (49.83%) ***	120 (58.54%) ***	72 (72.73%) ***
Postmenopausal	304 (64.54%)	138 (46.15%) ***	76 (37.07%) ***	23 (23.23%) ***
Unknown	10 (2.12%)	12 (4.01%)	9 (4.39%)	4 (4.04%)

### Quantitative BPE assessment

**Fig. 1:**
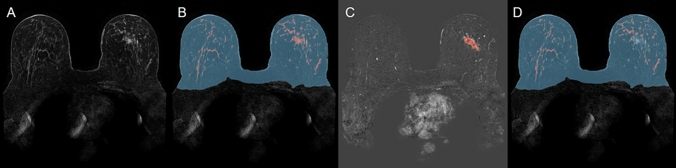
Illustration of fully automated mask generation for a representative patient. **A** Pre-contrast DCE-MRI scan. **B** Breast mask (blue) and FGT mask (red) generated using a 3D U-Net model. **C** Predicted tumor mask from the nnU-Net model overlaid on the signal enhancement map (first post-contrast image minus the pre-contrast image). **D** Refined FGT mask obtained by subtracting the tumor mask from the original FGT mask

Figure 1 illustrates the automatically generated masks used for quantitative BPE calculation based on DCE-MRI (Fig. 1A) from one representative patient. The breast and FGT segmentation masks are shown in Fig. 1B. Figure 1C, D displays the detected tumor voxels and the refined FGT mask obtained after removing tumor regions from the FGT mask.

**Fig. 2:**
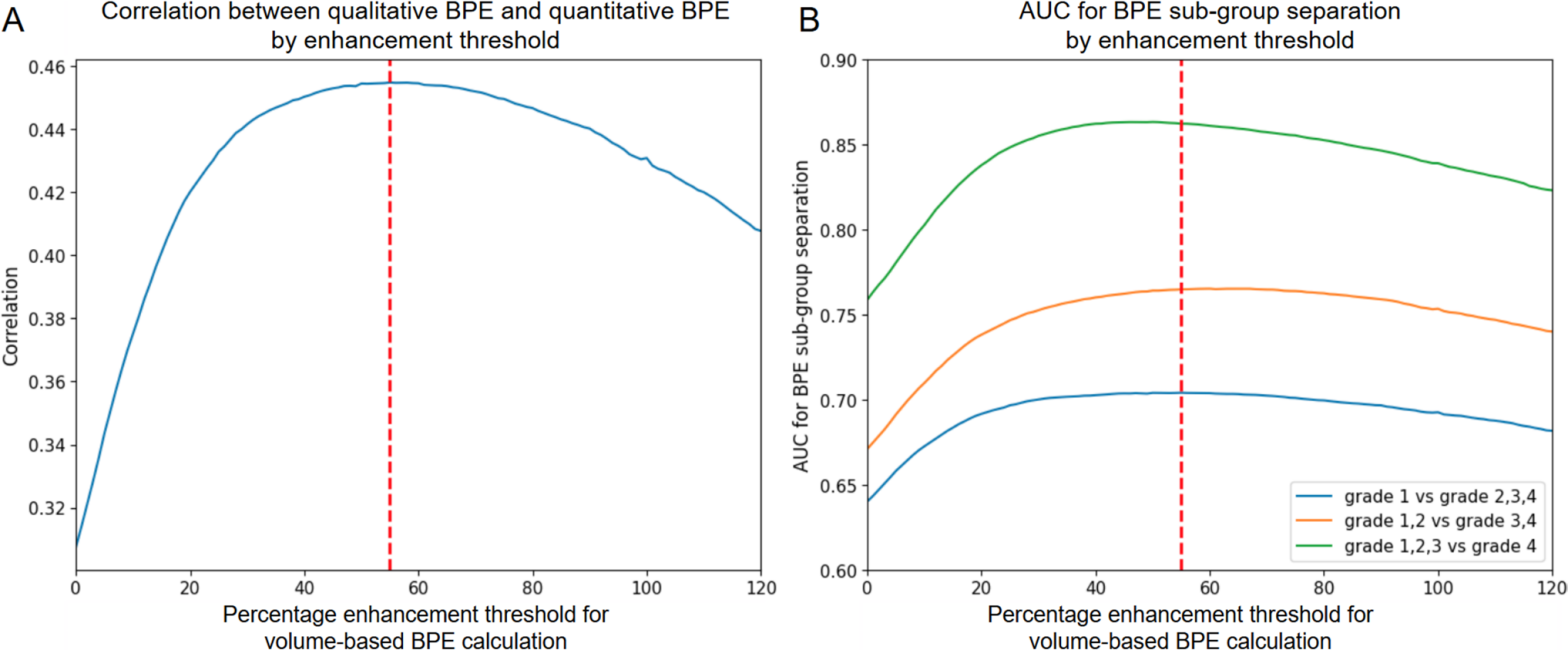
Correlation between qualitative BPE and quantitative BPE. **A** and the AUC of sub-group separation **B** by different enhancement thresholds for BPE calculation. The optimal correlation was achieved when enhancement threshold is 55% (denoted by the red vertical dash line), while the performance remains acceptable between 20 and 80% in terms of both correlation and AUC of sub-group separation

The quantitative BPE measurements demonstrated strong agreement with radiologist-defined BPE across a wide range of enhancement thresholds. Figure 2 shows the correlations between calculated and radiologist-defined BPE at different thresholds applied to the contralateral normal breast. Through multi-dimensional optimization, the highest correlation between the calculated and radiologist-defined BPE was obtained for enhancement threshold at 55%. When stratified by groups, the AUC separation between grade 1 vs. grades 2–4, grades 1–2 vs. grades 3–4, and grades 1–3 vs. grade 4 was 0.70, 0.76, and 0.86, respectively, for the optimal enhancement thresholds. AUCs were relatively invariant for enhancement threshold from 20 to 80%.

In a sub-analysis, BPE of the non-tumor FGT in the ipsilateral breast was evaluated (Supplementary Table 2). The correlation coefficient was lower, the optimal AUC was at a lower enhancement threshold, and the AUCs for separating different BPE groups were lowered compared to those used FGT of the contralateral breast.

In another sub-analysis, BPE was also evaluated using delayed post-contrast DCE-MRI (the 3rd post-contrast image). The AUC separation between grade 1 vs. grades 2–4, grades 1–2 vs. grades 3–4, and grades 1–3 vs. grade 4 was 0.69, 0.72, and 0.83, respectively, slightly lower than those obtained using the first post-contrast. The optimal enhancement threshold was 100%.

Intensity-based methods to calculate BPE were also evaluated (see Appendix). However, their performance indices were not superior to the volume-based methods, and they were not used for subsequent analyses.

### Prediction of OS and RFS

Analysis was performed to evaluate whether BPE predicted OS and RFS in the Duke-Breast-Cancer-MRI dataset for the entire cohort, those with NAC, and those with primary surgery (Table 2). The primary predictor was high (grade 3 and 4) vs. low (grade 2 and 1) grade. Other variables included age at breast cancer diagnosis, menopausal status, lymph node involvement, tumor stage, Nottingham histologic grade, molecular subtype, and neoadjuvant chemotherapy. Relative to low BPE grade, high BPE grade was associated with a better OS for the entire cohort (adjusted HR = 0.58 [0.34, 0.99]) and the primary surgery cohort (adjusted HR = 0.40 [0.16, 0.97]), but not the neoadjuvant chemotherapy cohort (adjusted HR = 0.73 [0.36, 1.47]). Relative to low BPE grade, high BPE grade was not associated with RFS in any cohort. When stratifying by age or menopausal status, BPE was not predictive of OS or RFS. The model for OS with HRs for all covariates is shown in Supplementary Table 3.

**Table 2:** Baseline BPE grade 3/4 vs. 1/2 hazard ratios (HR) for overall survival and recurrence-free survival. The primary predictor was grade 3/4 vs. 1/2 as predicted by quantitative BPE. Other variables included age at breast cancer diagnosis, menopausal status, lymph node involvement, tumor stage, Nottingham histologic grade, molecular subtype, and neoadjuvant chemotherapy. Only variables that were statistically significant in univariate analysis were included in multivariate models for each stratified sub-group. Each model did not adjust for the respective covariate that the cohort was being stratified by. *BPE* background parenchymal enhancement

Predicted quantitative BPE 3/4 vs. 1/2	Overall survival		Recurrence-free survival	
	Adjusted HR	*p* value	Adjusted HR	*p* value
Model includes entire cohort	0.58 [0.34, 0.99]	**0.046**	0.85 [0.59, 1.23]	0.39
Model includes neoadjuvant chemotherapy only	0.73 [0.36, 1.47]	0.37	0.80 [0.46, 1.39]	0.42
Model includes primary surgery only	0.40 [0.16, 0.97]	**0.043**	0.87 [0.54, 1.42]	0.57
Model includes post-menopausal only	0.48 [0.20, 1.15]	0.098	0.79 [0.47, 1.32]	0.37
Model includes pre-menopausal only	0.72 [0.32, 1.62]	0.42	0.99 [0.53, 1.82]	0.96
Model includes age > 52 only	0.53 [0.21, 1.30]	0.17	0.74 [0.42, 1.30]	0.30
Model includes age ≤ 52 only	0.67 [0.31, 1.42]	0.29	0.89 [0.51, 1.53]	0.66

Results were highlighed in bold if *p* <0.05

### Prediction of pCR

**Fig. 3:**
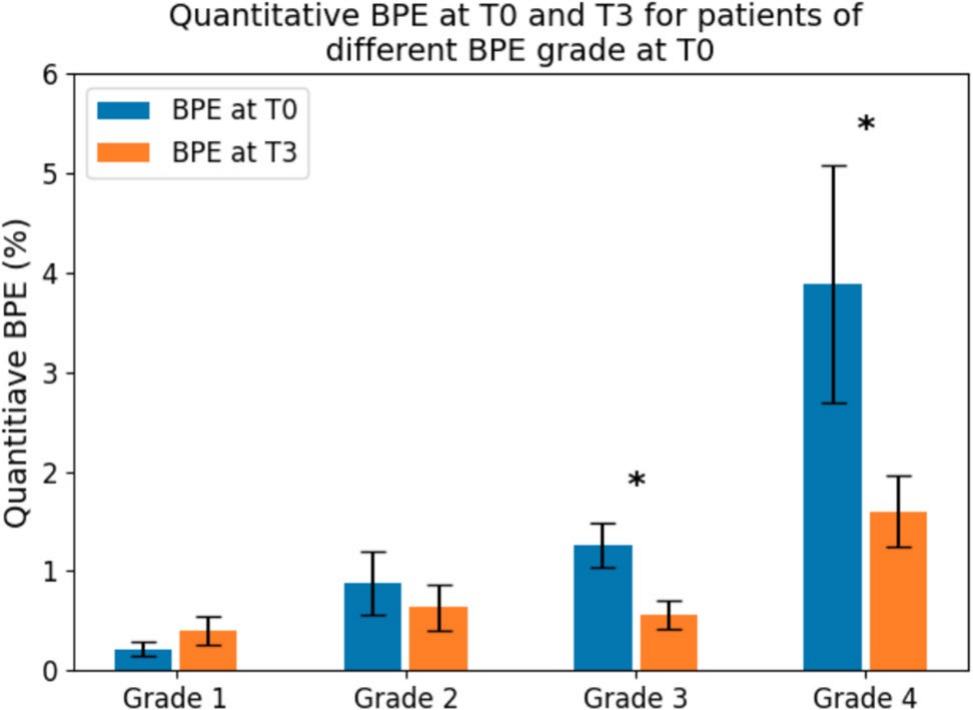
Quantitative BPE before (T_0_) and after (T_3_) NAC treatment from the I-SPY2 cohort whose pre- and post-treatment bilateral images are available (*n* = 114). Paired *t* test shows that patients originally graded as grade 3 and grade 4 show a significant decrease in quantitative BPE after treatment (*P* < 0.001 for grade 3 and *P* = 0.039 for grade 4)

BPE of post-NAC (T_3_) were generally lower than that of pre-NAC (T_0_) for all grades, with grade 3 and grade 4 at pre-NAC showing significant decreases in post-NAC BPE (*P* < 0.001 for grade 3 and *P* = 0.039 for grade 4) compared to pre-contrast BPE (I-SPY2 cohort, *n* = 114, Fig. 3). Analysis was performed to assess whether baseline BPE at T_0_ and change in BPE after NAC were associated with pCR (Table 3). In those with high baseline BPE (grade 3 and 4), a drop in BPE grade after NAC was predictive of pCR (aOR = 5.88 [1.03, 33.33]). In the low baseline BPE (grade 1 and 2) group, a drop in BPE grade after NAC was also predictive of pCR (aOR = 6.54 [1.26, 33.94]).

**Table 3:** Odds ratios (OR) for pathological complete response (pCR) for **A** baseline grade 3 or 4 and **B** baseline grade 1 or 2. *HER2* human epidermal growth factor receptor 2. *HR* hormone receptor. *FGT* fibroglandular tissue. *NAC* neoadjuvant chemotherapy

Baseline grade 3 or 4	Adjusted OR [95% CI]	*p* value
BPE 4 vs. 1/2/3 at T0	1.07 [0.23, 4.88]	0.93
Drop in quantitative BPE grade After NAC	5.88 [1.03, 33.33]	**0.046**

Baseline grade 1 or 2	Adjusted OR [95% CI]	*p* value
BPE 1 vs. 2/3/4 at T0	1.61 [0.38, 6.89]	0.52
Drop in quantitative BPE grade after NAC	6.54 [1.26, 33.94]	**0.025**
Increase in quantitative BPE grade after NAC	2.43 [0.41, 14.38]	0.33

Results were highlighed in bold if *p* <0.05

## Discussion

We implemented an automated BPE calculation using breast DCE-MRI and optimized the BPE thresholds to classify BPE grades, as well as used them to predict overall survival, recurrence-free survival and pathological complete response. The major findings are as follows: (1) The calculated BPE using the volume-based method on peaked contrast showed strong correlation with radiologist-defined BPE, with the best performance at 55% BPE enhancement threshold. (2) BPE from late post-contrast MRI yielded lower performance than BPE from peak post-contrast DCE-MRI. The intensity-based method to calculate BPE was inferior to the volume-based method. (3) Baseline BPE was independently associated with improved OS in the entire cohort (adjusted HR = 0.58 [0.34, 0.99]) and the primary surgery cohort (adjusted HR = 0.40 [0.16, 0.97]), but not the neoadjuvant chemotherapy cohort (adjusted HR = 0.73 [0.36, 1.47]). (4) BPE decreased post-neoadjuvant chemotherapy. A reduction in BPE after NAC was predictive of pCR (aOR = 5.88 [1.03, 33.33] for the high baseline BPE group and aOR = 6.54 [1.26, 33.94] for the low baseline BPE group). These findings underscore the potential utility of automatically calculated BPE as a prognostic imaging biomarker and a non-invasive indicator of treatment response, and survival outcomes in breast cancer.

### Profiles of patients with low and high BPE

The observation that patients with high BPE tend to be younger, premenopausal, more often Black, and more likely to present with triple-negative breast cancer aligns with prior work demonstrating that BPE reflects hormonal status and breast tissue vascularity, both of which are influenced by age, race, and tumor biology [24–26]. Elevated BPE has previously been associated with increased breast density and estrogen-related activity [3, 27]. Mahmoud et al. showed that Black women had higher BPE despite lower mammographic density compared to White women, differentiating BPE as a distinct biomarker separate from breast density [26]. Moreover, the association with triple-negative breast cancer—a subtype known for its aggressive behavior and lack of targeted hormonal therapies—may indicate that high BPE reflects not only systemic hormonal factors but also intrinsic tumor microenvironment characteristics such as angiogenesis and inflammation [28, 29].

### Calculated vs. radiologist-defined BPE

Automated BPE was highly correlated with radiologist-defined BPE categories, with optimal performance observed at a 55% enhancement threshold. This finding supports the validity of computational approaches for standardizing objective BPE assessment and aligns with prior efforts to develop reproducible, quantitative imaging biomarkers [30, 31]. Multiple studies have shown that quantitative methods for obtaining BPE yield higher AUC than subjective assessment of BPE [17, 18, 32]. Lam et al., for example, found the optimal thresholds at 10–40% on first post-contrast, achieving AUCs of 0.75 to 0.78 using various quantitative BPE methods [17]. Our findings are novel because we provided comprehensive optimization and evaluation of BPE calculation. Overall, while automated techniques offer superior scalability and reproducibility, further validation and standardization are needed to ensure reliable BPE quantification across clinical settings.

The relatively stable AUCs across a range of enhancement thresholds (20–80%) further suggest the robustness of this method, which could be helpful in clinical settings where contrast doses, acquisitions, and analysis methods could vary. The contralateral (non-tumor bearing) breast provides more reliable estimates of BPE than the ipsilateral breast, a finding supported by earlier studies indicating that tumor presence and therapy-induced changes can confound ipsilateral parenchymal measurements [24]. Nonetheless, the ability to estimate BPE from the ipsilateral breast with reasonable performance indices could have clinical utility in the event contralateral breast MRI is unavailable.

It is possible that delayed-phase DCE enhancement could provide better discriminating power than early enhancement, as the contrast has more time to diffuse into the extravascular space. However, delayed DCE enhancement has lower contrast-to-noise ratio compared to early-phase DCE enhancement. We observed early post-contrast DCE yielding better BPE prediction performance than delayed-phase DCE, consistent with prior kinetic analyses showing that peak enhancement occurs early in the post-contrast period and is most reflective of microvascular density and permeability [33–35].

### BPE and pCR

Longitudinal BPE analysis in the NAC cohort revealed significant reductions in BPE after NAC, particularly among patients with high baseline BPE. Our findings are consistent with Oh et al. who reported that the degree of qualitative BPE reduction post-NAC correlates with pathological tumor response to NAC [36]. These findings are also consistent with previous reports indicating that BPE is sensitive to anti-angiogenic, hormonal, and cytotoxic therapy, potentially serving as a surrogate marker for treatment-induced changes in vascularity, vascular permeability, and hormonal responsiveness [3, 37, 38]. These changes may reflect both direct effects on normal parenchymal tissue and systemic alterations in estrogen signaling pathways. Such findings are particularly relevant for monitoring response in hormone-sensitive tumors, but they also suggest that BPE could serve as a general indicator of therapy efficacy.

### BPE and survival

BPE was weakly associated with improved OS, after adjusting for potential confounders, such as age, tumor stage, nodal status, and molecular subtype, in the primary surgery cohort but not the NAC cohort. It is unclear why the NAC cohort showed no significant association. In our multivariable models (Supplementary Table 3), however, neither age nor menopausal status was significantly associated with overall survival, whereas BPE retained borderline statistical significance. This raises the possibility that BPE may capture aspects of the breast microenvironment, such as vascularity, stromal composition, or hormonal milieu that are not fully reflected by chronological age or menopausal status alone. Nonetheless, given the borderline statistical significance and potential for residual confounding, this association should be interpreted cautiously and warrants validation in independent cohorts. A possible explanation could be small sample size and further studies are needed. Prior studies have suggested that lower BPE may be associated with worse outcomes, potentially reflecting reduced hormonal sensitivity or a less vascular tumor microenvironment less amenable to therapy [3]. Two prior studies have investigated the correlation of quantitative BPE with prognosis. Moliere et al. quantitatively evaluated the BPE in a cohort of 102 patients with biopsy-proven breast cancer [37]. They calculated BPE as the total volume of the enhancing voxels over the FGT region that had an enhancement ratio of greater than or equal to 20% and found that quantitative BPE post-NAC, but not pre-NAC BPE, is predictive of recurrence and disease-free survival, independent of pCR (102 biopsy-proven invasive breast cancer). Rella et al. found that baseline BPE, final BPE, and change in BPE had no association with disease-free survival or OS in a 228 patient cohort with invasive breast cancer [39], and no association of baseline, final, or change in BPE with disease-free survival or OS. Their BPE calculation was simplistic which used the manually drawn ROI mean signal intensity (SI) of a manually drawn ROI and did not incorporate automated segmentation or a percent threshold, unlike some other quantitative studies. Together these findings underscore the prognostic value of this imaging feature.

## Limitations

The strengths and novelties of this study included large sample size, automated breast segmentation, automated FGT segmentation, optimization of performance indices for different BPE grade classification, and multiple predictions, including the prognostic values of baseline BPE and changes in BPE for predicting complete pathological response, overall survival, and recurrence-free survival, with comparison with known confounders.

This study has several limitations. First, qualitative BPE categories were determined by consensus review, precluding formal assessment of inter-reader variability. Second, while automated quantification of BPE provides objective measurements, the approach depends on accurate segmentation of FGT, which can be affected by image quality, motion artifacts, and variation in MRI acquisition protocols across institutions. Third, although the use of a large, diverse dataset strengthens generalizability, the retrospective nature of the study limits the ability to infer causal relationships, and the findings may be influenced by unmeasured confounding variables. Moreover, the association between BPE reduction and pCR was only significant in patients with high baseline BPE, indicating that BPE may not serve as a universal predictor of treatment response. Lastly, while this study used early and delayed post-contrast images to evaluate BPE performance, dynamic contrast-enhanced imaging parameters (e.g., temporal resolution and kinetic curve modeling) were not fully explored and may provide additional insight into vascular dynamics and treatment response. We did not investigate BPE and cancer risk [18, 40]. We did not compare our model predictions with prior studies predicting pCR, RFS, and OS without including BPE [41–43]. Future prospective studies with harmonized imaging protocols and longitudinal follow-up are needed to validate these findings and support clinical integration.

## Conclusion

Automated calculation of BPE grade is consistent with radiologist-defined BPE grades. Calculated BPE grade showed prognostic value for recurrence-free and overall survival. Changes in BPE during neoadjuvant chemotherapy were predictive of treatment response. Overall, these findings support the incorporation of automated BPE quantification into clinical workflows and research protocols aimed at improving individualized risk assessment, therapy monitoring, and long-term outcome prediction in breast cancer patients.

## Supplementary Information

Below is the link to the electronic supplementary material.

Supplementary file1 (DOCX 23 kb)

## Data Availability

I-SPY2: https:/www.cancerimagingarchive.net/collection/ispy2; Duke Breast Cancer MRI: https://www.cancerimagingarchive.net/collection/duke-breast-cancer-mri/
