# Supplementary material for: Automated calculation of background parenchymal enhancement as a biomarker of treatment responses and recurrence-free survival in breast cancer
Source: Breast Cancer Res Treat. 2026 Mar 24;216(3):35. doi: 10.1007/s10549-026-07941-5 (PMC13009020; doi:10.1007/s10549-026-07941-5)
Supplement: Supplementary file 1 — Supplementary file1 (DOCX 23 kb) [file 10549_2026_7941_MOESM1_ESM.docx]

**Supplementary Table 1** Baseline demographics of patients in the Duke-Breast-Cancer-MRI cohort used for survival analysis grouped by (**A**) BPE grade and (**B)** treatment types for high and low BPE grade. *SD* standard deviation. *IQR* interquartile range. *HR* hormone receptor. *HER2* human epidermal growth factor receptor 2. **p* < 0.05, ***p* < 0.01, and ****p* < 0.001 as compared to grade 1 in A and grade 1/2 in B

| A) | Qualitative background parenchymal enhancement grade | | | |
| --- | --- | --- | --- | --- |
|  | 1 (*n*=435) | 2 (*n*=245) | 3 (*n*=161) | 4 (n=81) |
| Age |  |  |  |  |
| Age, mean ± SD | 55.22 ± 11.58 | 52.53 ± 10.53 ** | 50.50 ± 10.13 *** | 46.88 ± 9.77 *** |
| Age, median [IQR] | 55.06 [47.94, 63.29] | 51.69 [45.45, 59.80] ** | 49.23 [43.89, 56.83] *** | 45.72 [41.50, 51.20] *** |
| Race/Ethnicity |  |  |  |  |
| White | 311 (71.49%) | 168 (68.57%) | 116 (72.05%) | 56 (69.14%) |
| Black or African American | 92 (21.15%) | 57 (23.27%) | 33 (20.50%) | 21 (25.93%) |
| Asian | 5 (1.15%) | 6 (2.45%) | 2 (1.24%) | 1 (1.23%) |
| Hispanic or Latino | 9 (2.07%) | 4 (1.63%) | 3 (1.86%) | 2 (2.47%) |
| American Indian or Alaska Native | 13 (2.99%) | 4 (1.63%) | 5 (3.11%) | 1 (1.23%) |
| Native Hawaiian or Pacific Islander | 1 (0.23%) | 2 (0.82%) | 1 (0.62%) | 0 (0.00%) |
| Multi-Racial | 4 (0.92%) | 4 (1.63%) | 1 (0.62%) | 0 (0.00%) |
| Molecular Subtype |  |  |  |  |
| HR+ HER2− | 284 (65.29%) | 154 (62.86%) | 105 (65.22%) | 52 (64.20%) |
| HR+ HER2+ | 51 (11.72%) | 30 (12.24%) | 14 (8.70%) | 9 (11.11%) |
| HR− HER2+ | 25 (5.75%) | 18 (7.35%) | 14 (8.70%) | 2 (2.47%) |
| HR−/HER2− (Triple Negative) | 75 (17.24%) | 43 (17.55%) | 28 (17.39%) | 18 (22.22%) |
| Menopausal Status |  |  |  |  |
| Premenopausal | 141 (32.41%) | 119 (48.57%) *** | 89 (55.28%) *** | 58 (71.60%) *** |
| Postmenopausal | 289 (66.44%) | 121 (49.39%) *** | 67 (41.61%) *** | 22 (27.16%) *** |
| Unknown | 5 (1.15%) | 5 (2.04%) | 5 (3.11%) | 1 (1.23%) |
| Outcomes at Last Follow-Up |  |  |  |  |
| All-Cause Mortality | 33 (7.59%) | 17 (6.94%) | 10 (6.21%) | 2 (2.47%) |
| All-Cause Mortality or Recurrence | 57 (13.10%) | 30 (12.24%) | 24 (14.91%) | 7 (8.64%) |

| B) | Neoadjuvant Chemotherapy | | Primary Surgery | |
| --- | --- | --- | --- | --- |
|  | BPE grade 1/2 (*n*=199) | BPE grade 3/4 (*n*=93) | BPE grade 1/2 (*n*=481) | BPE grade 3/4 (*n*=149) |
| Age |  |  |  |  |
| Age, mean ± SD | 50.25 ± 11.47 | 45.68 ± 8.96 *** | 55.91 ± 10.79 | 51.53 ± 10.21 *** |
| Age, median [IQR] | 49.95 [42.00, 59.21] | 45.35 [41.12, 51.20] *** | 55.67 [48.82, 63.24] | 49.90 [44.23, 57.56] *** |
| **Race/Ethnicity** |  |  |  |  |
| White | 118 (59.30%) | 60 (64.52%) | 361 (75.05%) | 112 (75.17%) |
| Black or African American | 65 (32.66%) | 26 (27.96%) | 84 (17.46%) | 28 (18.79%) |
| Asian | 5 (2.51%) | 2 (2.15%) | 6 (1.25%) | 1 (0.67%) |
| Hispanic or Latino | 4 (2.01%) | 3 (3.23%) | 9 (1.87%) | 2 (1.34%) |
| American Indian or Alaska Native | 2 (1.01%) | 1 (1.08%) | 15 (3.12%) | 5 (3.36%) |
| Native Hawaiian or Pacific Islander | 2 (1.01%) | 1 (1.08%) | 1 (0.21%) | 0 (0.00%) |
| Multi-racial | 3 (1.51%) | 0 (0.00%) | 5 (1.04%) | 1 (0.67%) |
| Molecular subtype |  |  |  |  |
| HR+ HER2− | 82 (41.21%) | 41 (44.09%) | 356 (74.01%) | 116 (77.85%) |
| HR+ HER2+ | 40 (20.10%) | 14 (15.05%) | 41 (8.52%) | 9 (6.04%) |
| HR− HER2+ | 22 (11.06%) | 8 (8.60%) | 21 (4.37%) | 8 (5.37%) |
| HR−/HER2− (Triple Negative) | 55 (27.64%) | 30 (32.26%) | 63 (13.10%) | 16 (10.74%) |
| Menopausal status |  |  |  |  |
| Premenopausal | 96 (48.24%) | 70 (75.27%) *** | 164 (34.10%) | 77 (51.68%) *** |
| Postmenopausal | 102 (51.26%) | 23 (24.73%) *** | 308 (64.03%) | 66 (44.30%) *** |
| Unknown | 1 (0.50%) | 0 (0.00%) | 9 (1.87%) | 6 (4.03%) |
| Adjuvant therapies |  |  |  |  |
| Adjuvant chemotherapy | 151 (34.71%) | 102 (41.63%) | 56 (34.78%) | 27 (33.33%) |
| Adjuvant endocrine therapy | 280 (64.37%) | 160 (65.31%) | 107 (66.46%) | 52 (64.20%) |
| Adjuvant radiation therapy | 299 (68.74%) | 151 (61.63%) | 107 (66.46%) | 57 (70.37%) |
| Adjuvant anti-HER2 Therapy | 57 (13.10%) | 44 (17.96%) | 27 (16.77%) | 10 (12.35%) |
| Outcomes at last follow-up |  |  |  |  |
| All-cause mortality | 23 (11.56%) | 8 (8.60%) | 27 (5.61%) | 4 (2.68%) |
| All-cause mortality or recurrence | 35 (17.59%) | 16 (17.20%) | 52 (10.81%) | 15 (10.07%) |

**Supplementary Table 2** Correlation between calculated BPE and radiologist-defined ground truths, optimal enhancement thresholds, and the AUC of BPE sub-group separation using BPE measurements derived from the contralateral breast and the ipsilateral breast under the optimal enhancement threshold. Note: tumor voxels were excluded for the ipsilateral breast images.

|  | Contralateral breast | Ipsilateral breast |
| --- | --- | --- |
| Best correlation | 0.45 | 0.37 |
| Enhancement threshold | 55% | 32% |
| AUC: grade 1 vs. grades 2–4 | 0.704 | 0.662 |
| AUC: grades 1–2 vs. grades 3–4 | 0.765 | 0.709 |
| AUC: grades 1–3 vs. grade 4 | 0.862 | 0.827 |

**Supplementary Table 3** Baseline BPE grade 3/4 vs. 1/2 hazard ratios (HR) for overall survival. The primary predictor was grade 3/4 vs. 1/2 as predicted by quantitative BPE. Other variables included age at breast cancer diagnosis, menopausal status, lymph node involvement, tumor stage, Nottingham histologic grade, molecular subtype, and neoadjuvant chemotherapy. BPE as well as other variables that were statistically significant in univariate analysis were included in multivariate models. BPE, background parenchymal enhancement.

| Predictor | Overall Survival | | | |
| --- | --- | --- | --- | --- |
|  | Univariate | | Multivariate | |
|  | Unadjusted HR | *p* value | Adjusted HR | *p* value |
| Predicted quantitative BPE 3/4 vs. 1/2 | 0.62 [0.36, 1.06] | 0.079 | 0.58 [0.34, 0.99] | **0.046** |
| Age at cancer diagnosis (Years) | 1.01 [0.99, 1.03] | 0.41 | **−** | **−** |
| Postmenopausal vs. premenopausal | 1.32 [0.79, 2.20] | 0.29 | **−** | **−** |
| Lymph node invasion | 2.32 [1.40, 3.86] | **<0.005** | 2.16 [1.27, 3.70] | **<0.005** |
| T stage ≥ 2 | 2.35 [1.34, 4.11] | **<0.005** | 1.90 [1.06, 3.41] | **0.031** |
| Nottingham histologic grade 3 vs. 1 | 1.77 [1.07, 2.91] | **0.026** | 1.13 [0.43, 3.01] | 0.80 |
| Nottingham histologic grade 2 vs. 1 | 0.79 [0.48, 1.31] | 0.36 | 1.15 [0.46, 2.84] | 0.76 |
| ER+/HER2+(±PR) vs. ER+/HER2− (±PR) | 0.43 [0.26, 0.72] | **<0.005** | 1.65 [0.56, 4.87] | 0.36 |
| ER−/HER2+(±PR) vs. ER+/HER2− (±PR) | 0.98 [0.36, 2.70] | 0.97 | 1.79 [0.45, 7.15] | 0.41 |
| ER−/HER2−(PR−) vs. ER+/HER2− (±PR) | 3.62 [2.19, 5.97] | **<0.005** | 5.37 [1.85, 15.58] | **<0.005** |
| Neoadjuvant Chemotherapy vs. Primary Surgery | 2.14 [1.30, 3.53] | **<0.005** | 1.24 [0.70, 2.20] | 0.46 |

**Intensity-based BPE calculation**

The formula for two additional intensity-based BPE calculations is as follows:

Method A:

$${BPE}_{i,k}^{A}=\frac{\sum_{j\in FGT} S_{i,j}-\sum_{j\in FGT} S_{0,j}}{\sum_{j\in FGT} S_{0,j}}\times100\%$$

where $S_{0,j}$ denotes the signal intensity of voxel j on the pre-contrast image and $S_{i,j}$ denotes the signal intensity of the same voxel on the i post-contrast image. This method emphasizes the overall signal enhancement of all FGT.

Method B:

$${BPE}_{i,k}^{B}=\frac{1}{\left| FGT \right|}\sum_{j\in FGT} {Top}_{k}\left( \frac{S_{i,j}-S_{0,j}}{S_{0,j}} \right)\times100\%$$

where $S_{0,j}$ denotes the signal intensity of voxel j on the pre-contrast image and $S_{i,j}$ denotes the signal intensity of the same voxel on the i post-contrast image. This method represents average FGT enhancement with top k percent enhancement thresholding. In our dataset, maximum Spearman correlation between qualitative BPE and quantitative BPE was achieved when *k*=77%. The AUC of sub-group separation is shown in Supplemental Table 3

**Supplementary Table 4** Correlation between qualitative BPE quantitative BPE and AUC of BPE sub-group separation using the volume-based BPE calculation from the main text of the article and two intensity-based BPE calculations mentioned above on the contralateral side breast.

| Quantitative BPE method | Volume-based method | Intensity-based method A | Intensity-based method B |
| --- | --- | --- | --- |
| Best correlation | 0.45 | 0.47 | **0.48** |
| AUC: grade 1 vs. grades 2–4 | 0.704 | **0.736** | 0.733 |
| AUC: grades 1–2 vs. grades 3–4 | **0.765** | 0.743 | 0.755 |
| AUC: grades 1–3 vs. grade 4 | **0.862** | 0.831 | 0.844 |
